# Associated Factors of Cervical Spinal Cord Injury Without Radiographic Evidence of Trauma: A Retrospective Study

**DOI:** 10.7759/cureus.80006

**Published:** 2025-03-04

**Authors:** Kenta Nakamura, Masatoshi Teraguchi, Tomonori Nakata, Takahiro Kozaki, Masanari Takami, Keiji Nagata, Yuyu Ishimoto, Kentaro Ueda, Shigeaki Inoue, Hiroshi Yamada

**Affiliations:** 1 Department of Orthopaedic Surgery, Wakayama Medical University, Wakayama, JPN; 2 Department of Emergency and Critical Care Medicine, Wakayama Medical University, Wakayama, JPN

**Keywords:** associated factors, cervical canal stenosis, diffuse idiopathic skeletal hyperostosis (dish), ossification of the posterior longitudinal ligament, spinal cord injury without radiographic evidence of trauma

## Abstract

Background

Spinal cord injury without radiographic evidence of trauma (SCIWORET) represents a significant portion of cervical spinal cord injuries (SCIs), particularly in elderly patients. Despite its clinical significance, the risk factors and their interactions remain poorly understood. This study aimed to identify key predictors of SCIWORET by analyzing anatomical and biomechanical factors in cervical SCI patients at a single tertiary emergency medical center.

Methods

We retrospectively analyzed consecutive patients with C3-C7 cervical SCI between April 2011 and November 2023. All patients underwent standardized neurological examination and comprehensive imaging studies, including whole-spine computed tomography (CT) and cervical magnetic resonance imaging (MRI). SCIWORET was defined as a neurological deficit without fracture, dislocation, or discoligamentous complex (DLC) injuries on imaging. We evaluated the presence of cervical canal stenosis (CCS), ossification of the posterior longitudinal ligament (OPLL), and diffuse idiopathic skeletal hyperostosis (DISH) using standardized criteria. Two experienced orthopedic surgeons independently assessed all imaging studies. Univariate logistic regression analysis was first examined to identify the associated factor of SCIWORET. We evaluated multicollinearity using variance inflation factors (VIFs) and correlation coefficients between CCS, OPLL, and DISH. Furthermore, multivariate logistic regression analysis was conducted to identify independent predictors of SCIWORET.

Results

Among the study population, 203 of 348 patients (58.3%) were diagnosed with SCIWORET. CCS was present in 174 of 348 patients (50.0%), with a significantly higher prevalence in the SCIWORET group (174 of 203 patients, or 85.7%) compared to the SCI with fracture group (78 of 145 patients, or 53.8%). OPLL was identified in 68 of 203 patients (33.5%), showing a higher prevalence in the SCIWORET group versus the SCI with fracture group (24 of 145 patients, or 16.6%). Regarding VIF, CCS, cervical OPLL, and DISH were 1.07, 1.12, and 1.19, respectively. Correlation analysis showed weak associations: CCS and DISH (r = 0.11), CCS and cervical OPLL (r = 0.25), and DISH to cervical OPLL (r = 0.33). Multivariate logistic regression analysis revealed that CCS (odds ratio (OR): 4.91, 95% CI: 2.78-8.70, p < 0.0001) and cervical OPLL (OR: 1.83, 95% CI: 1.01-3.29, p < 0.05) were independent predictors of SCIWORET, respectively.

Conclusions

CCS, with or without cervical OPLL, are independent predictors of SCIWORET. These findings emphasize the importance of comprehensive spinal evaluation in trauma patients, particularly in those presenting with neurological symptoms following low-energy trauma. Our results suggest that patients with pre-existing CCS or OPLL may be at increased risk for SCI, even in the absence of obvious radiographic abnormalities.

## Introduction

Cervical spinal cord injury without radiographic evidence of trauma (SCIWORET) represents a unique and challenging clinical entity in spine surgery and emergency medicine [1]. First described by Allen in 1908, SCIWORET is characterized by neurological deficits in the absence of obvious fractures or dislocations on conventional imaging [2]. The reported incidence ranges from 52% to 54% of all cervical spinal cord injuries (SCIs), with higher rates observed [3,4]. In Japan, similar incidence rates have been reported, ranging from 38% to 60% [5,6]. These variations in incidence rates appear to be influenced by multiple factors, including ethnicity, environmental conditions, and age. The clinical significance of SCIWORET has grown considerably in recent decades, parallel to global population aging [3-7]. Elderly patients, particularly those with pre-existing cervical conditions, appear to be at increased risk for this condition [5]. The mechanisms underlying SCIWORET are complex and likely multifactorial, involving both anatomical and biomechanical factors that may predispose individuals to SCI even in the absence of high-energy trauma [6].

Several structural factors have been proposed as potential contributors to SCIWORET. Cervical canal stenosis (CCS) has been identified as a significant risk factor, as the narrowed spinal canal provides less space for cord movement and increases vulnerability to injury [8,9]. Ossification of the posterior longitudinal ligament (OPLL), particularly prevalent in Asian populations, may further compromise the space available for the spinal cord and alter spine biomechanics [10]. Additionally, diffuse idiopathic skeletal hyperostosis (DISH), characterized by flowing ossification along the spine, may influence spinal mobility and stress distribution patterns [11-13].

Despite these observations, the relative importance and potential interactions between these factors remain incompletely understood. Previous studies have focused on individual risk factors rather than their combined effects [3-9]. However, previous studies have not comprehensively investigated SCIWORET through the combined assessment of whole-spine computed tomography (CT), cervical magnetic resonance imaging (MRI), injury mechanisms, and neurological severity simultaneously.

This study aimed to comprehensively investigate the factors associated with SCIWORET in cervical SCI patients, with particular attention to the roles of CCS, cervical OPLL, and DISH.

## Materials and methods

Study design and ethics

This retrospective observational study was conducted at a tertiary emergency medical center that manages over 200 severe trauma cases (Injury Severity Score ≥ 16) annually. The requirement for informed consent was waived due to the retrospective design. All research procedures were conducted in accordance with the ethical standards of the 1964 Declaration of Helsinki and its later amendments.

Study population and patient selection

This study analyzed consecutive patients diagnosed with cervical SCI at our institution between April 2011 and November 2023. From an initial cohort of 516 patients, we conducted a careful selection process based on predetermined criteria. Patients were eligible for inclusion if they had sustained cervical SCI at C3-C7 levels, as confirmed by both clinical examination and imaging studies. Additionally, we required complete imaging documentation, including whole-spine CT and cervical MRI, to ensure a comprehensive evaluation of spinal pathology. Neurological status at emergency department admission needed to be reliably classifiable according to the American Spinal Injury Association (ASIA) impairment scale to ensure accurate baseline assessment [14].

We excluded patients with injuries at C1-C2 levels due to the distinct biomechanical and clinical characteristics of upper cervical injuries. Cases with incomplete imaging studies were also excluded to maintain consistency in the radiological assessment. Furthermore, patients whose neurological examination was deemed unreliable were excluded to ensure data integrity. This included patients with consciousness disturbance, severe sedation, delirium, intubation with the inability to participate in a complete neurological assessment, and significant cognitive impairment that would prevent reliable examination. This exclusion was necessary to ensure accurate classification of neurological status according to the ASIA impairment scale. After applying these criteria, our final study population comprised 348 patients (267 males and 81 females), with a mean age of 65.5 ± 15.1 years.

Clinical assessment and data collection

All patients underwent a standardized neurological examination upon arrival at the emergency department. Neurological status was evaluated using the ASIA impairment scale by experienced spine surgeons. The following clinical data were collected: age, gender, mechanism of injury (categorized as high-energy or low-energy trauma), neurological status, and associated injuries.

Neurological assessment

The ASIA impairment scale was used to classify neurological status at admission. This standardized assessment tool categorizes impairment into five grades: Grade A, complete SCI with no sensory or motor function preserved below the neurological level, including the sacral segments S4-S5; Grade B, incomplete injury with preserved sensory but no motor function below the neurological level; Grade C, incomplete injury with preserved motor function below the neurological level, with most key muscles having a strength grade less than 3/5; Grade D, incomplete injury with preserved motor function below the neurological level, with most key muscles having a strength grade of 3/5 or greater; and Grade E, normal sensory and motor functions.

In this study, we classified trauma mechanisms into high-energy trauma and low-energy trauma based on established criteria from previous literature [15]. High-energy trauma was defined as motor vehicle accidents, pedestrian or cyclist injuries, falls from a height greater than 2 m, direct impact from falling heavy objects, and high-velocity sports injuries. On the other hand, falls on flat surfaces were classified as low-energy trauma.

Imaging protocol and analysis

All patients underwent standardized imaging protocols upon admission. Whole spine CT was performed using a 128-slice multidetector CT scanner (SOMATOM Definition AS; Siemens Healthcare, Forchheim, Germany) with the following parameters: 120 kV, 380 mA, and 0.6 mm slice thickness. MRI was conducted using a 1.5-T scanner (Achieva; Philips Healthcare, Amsterdam, the Netherlands) with a standardized protocol.

Radiological definitions and assessment

SCIWORET was defined as the presence of neurological deficit without evidence of fracture, dislocation, or discoligamentous complex (DLC) injuries on CT or MRI. In this study, we specifically used the definition of SCIWORET, which excludes not only bony injuries but also all DLC injuries visible on MRI. However, not all patients underwent functional X-ray at admission due to preventing exacerbation of neurological deficits.

CCS was assessed on T2-weighted sagittal MRI images using a validated grading system: Grade 1, no compression of the spinal cord with subarachnoid space absent; Grade 2, compression of less than one-third of the spinal cord; Grade 3, compression of more than one-third but less than two-thirds of the spinal cord; and Grade 4, compression of more than two-thirds of the spinal cord. CCS was defined as Grade 2 or higher at the most severely affected intervertebral disc level [16]. CCS was considered present when Grade 2 or higher compression was observed at any cervical level. OPLL was evaluated according to the Japanese Ministry of Health and Welfare criteria, using CT imaging [17]. DISH was diagnosed based on the Resnick criteria, requiring flowing ossification along at least four consecutive vertebral bodies, with preservation of disc height and absence of degenerative disc changes [11].

All imaging studies were independently evaluated by two experienced orthopaedic surgeons (K.N. and M.T.), who were blinded to clinical information. Disagreements were resolved by consensus.

Statistical analysis

Statistical analyses were performed using JMP version 16-1 (SAS Institute Inc., Cary, NC, USA). Continuous variables are presented as means ± standard deviations, and categorical variables as numbers and percentages. Between-group comparisons were performed using Student's t-test for continuous variables and Chi-square or Fisher's exact test for categorical variables.

Univariate logistic regression analysis was first examined to identify the associated factor of SCIWORET. We evaluated multicollinearity using variance inflation factors (VIFs) and correlation coefficients between CCS, OPLL, and DISH. Furthermore, multivariate logistic regression analysis was conducted to identify independent predictors of SCIWORET.

Results are presented as odds ratios (ORs) with 95% confidence intervals (CI). Inter-observer reliability for radiological assessments was evaluated using weighted kappa coefficients, with 95% CI. Statistical significance was set at p < 0.05 for all analyses.

Reliability assessment

Inter-observer reliability analysis demonstrated substantial to excellent agreement for all radiological parameters (κ > 0.78). The highest agreement was observed for OPLL assessment (κ = 0.91, 95% CI: 0.82-0.92), followed by DISH evaluation (κ = 0.85, 95% CI: 0.78-0.92), and CCS grading (κ = 0.78, 95% CI: 0.71-0.85).

## Results

Patient demographics and clinical characteristics

Among the 348 patients included in the final analysis, there were 267 males (76.7%) and 81 females (23.3%), with a mean age of 65.5 ± 15.1 years. SCIWORET was identified in 203 patients (58.3%). The mechanism of injury was categorized as low-energy trauma in 81 cases (23.3%) and high-energy trauma in 267 cases (76.7%). The distribution of injury mechanisms is shown in Table 1.

**Table 1:** Characteristics of the study group. SCIWORET, spinal cord injury without radiographic evidence of trauma; DISH, diffuse Idiopathic skeletal hyperostosis; OPLL, ossification of posterior longitudinal ligament; CCS, cervical canal stenosis; ASIA, American Spinal Injury Association

Characteristic	Value
No. of cases (male/female)	348 (267/81)
Age, years	65.5 ± 15.1
Prevalence of SCIWORET (%)	145 (41.7%)
Case by ASIA impairment scale (%)	
Grade A	33 (9.5%)
Grade B	40 (11.5%)
Grade C	116 (33.3%)
Grade D	63 (18.1%)
Grade E	96 (27.6%)
Mechanical of injury (%)	
Traffic accident	95 (35.3%)
Fall from height	123 (39.0%)
Falling of heavy objects,	8 (2.7%)
Diving	6 (2.0%)
Fall on level surface	81 (21.0%)
Others	35 (10.1%)
Prevalence of DISH (%)	160 (37.7%)
Prevalence of cervical OPLL (%)	92 (23.4%)
Prevalence of CCS (%)	252 (73.0%)

Severe paralysis (ASIA Grades A and B) was observed in 73 patients (21.0%). According to Table 2, severe paralysis (Grades A and B) was observed in 40 patients (19.7%) in the SCIWORET group, compared to 34 patients (23.5%) in the SCI with fracture group (p = 0.4). The complete distribution of ASIA impairment scales is presented in Table 2.

**Table 2:** Characteristics between presence of spinal cord injury without radiographic evidence of trauma and spinal cord injury with fracture. SCIWORET, spinal cord injury without radiographic evidence of trauma; DISH, Diffuse Idiopathic skeletal hyperostosis; OPLL, ossification of posterior longitudinal ligament; CCS, cervical canal stenosis; ASIA, American Spinal Injury Association

Characteristic	SCIWORET group	SCI with fracture	p-value
No. of cases (male/female)	203 (159/44)	145 (108/37)	0.4
Age, years	66.0 ± 13.7	64.8 ± 16.9	0.46
Case by ASIA impairment scale (%)			
Severe paralysis (Grades A and B)	40 (19.7%)	34 (23.5%)	0.4
Non-severe paralysis (Grades C-E)	163 (80.3%)	111 (76.5%)	
Mechanical of injury (%)			
High energy (traffic accident, high fall, sports, etc.)	146 (72.3%)	121 (83.4%)	<0.05
Low energy (fall on level surface)	56 (27.6%)	25 (17.2%)	
Prevalence of cervical OPLL (%)	68 (33.5%)	24 (16.6%)	<0.001
Prevalence of CCS (%)	174 (85.7%)	78 (53.8%)	<0.0001
Prevalence of DISH (%)	98 (48.3%)	62 (42.8%)	0.31

Cervical OPLL was identified in 68 patients, showing a higher prevalence in the SCIWORET group (33.5% vs. 16.6%, p < 0.001). CCS was present in 174 patients, with a significantly higher prevalence in the SCIWORET group compared to the SCI with fracture group (85.7% vs. 53.8%, p < 0.0001). DISH was observed in 98 patients, with a non-significant difference between groups (48.3% vs. 42.8%, p = 0.31).

Univariate analysis revealed several significant factors associated with SCIWORET: cervical OPLL (OR: 2.99, 95% CI: 1.57-5.70, p < 0.001), CCS (OR: 5.48, 95% CI: 3.15-9.52, p < 0.0001), and high-energy trauma (OR: 0.52, 95% CI: 0.31-0.86, p < 0.05 (Table 3).

**Table 3:** Univariate analysis for associated factor to spinal cord injury without radiographic evidence of trauma. OR, odds ratio; CI, confidential interval; DISH, diffuse Idiopathic skeletal hyperostosis; OPLL, ossification of posterior longitudinal ligament; CCS, cervical canal stenosis

Characteristic	OR	95% CI	p-value
Older age (>65 years)	1.44	0.90-2.29	0.13
Male vs. female	1.27	0.74-2.14	0.38
High energy trauma	0.49	0.26-0.91	<0.05
Presence of cervical OPLL	2.99	1.57-5.70	<0.001
Presence of CCS	5.48	3.15-9.52	<0.0001
Presence of DISH	1.25	0.81-1.92	0.31

Regarding VIF, CCS, cervical OPLL, and DISH were 1.07, 1.12, and 1.19, respectively, indicating acceptable levels of correlation that do not substantially affect our regression estimates. Correlation analysis showed weak associations: CCS and DISH (r = 0.11), CCS and cervical OPLL (r = 0.25), and DISH and cervical OPLL (r = 0.33).

Multivariate logistic regression analysis revealed that CCS (OR: 4.91, 95% CI: 2.78-8.70, p < 0.0001) and cervical OPLL (OR: 1.83, 95% CI: 1.01-3.29, p < 0.05) were independent predictors of SCIWORET. High-energy trauma showed a negative association but was not statistically significant in the multivariate analysis (OR: 0.72, 95% CI: 0.41-1.29, p = 0.27). DISH showed no significant association in the multivariate analysis (OR: 1.00, 95% CI: 0.59-1.70, p = 0.99) (Table 4).

**Table 4:** Multivariate analysis for associated factor to spinal cord injury without radiographic evidence of trauma. OR, odds ratio; CI, confidential interval; DISH, diffuse Idiopathic skeletal hyperostosis; OPLL, ossification of posterior longitudinal ligament; CCS, cervical canal stenosis

Characteristic	OR	95% CI	p-value
Older age (>65 years)	1.35	0.79-2.28	0.27
Male vs. female	1.13	0.63-2.03	0.68
High energy trauma	0.72	0.41-1.29	0.27
Presence of cervical OPLL	1.83	1.01-3.29	<0.05
Presence of CCS	4.91	2.78-8.70	<0.0001
Presence of DISH	1.00	0.59-1.70	0.99

## Discussion

This study provides comprehensive evidence regarding the factors associated with SCIWORET in cervical SCI patients. Our findings demonstrate the complex interplay between anatomical factors and injury mechanisms in the development of this challenging clinical entity.

The strong association between CCS and SCIWORET (OR: 4.91, 95% CI: 2.78-8.70) represents the most significant finding of our study. This relationship aligns with previous research by Koyanagi et al. [5], who reported that pre-existing stenosis increases vulnerability to SCI, even in minor trauma. The reduced space available for cord movement in stenotic canals may explain the increased susceptibility to injury without obvious radiographic abnormalities [5,6]. Therefore, some studies have emphasized the significance of prompt diagnosis of SCIWORET [5-9].

Our finding that CCS was present in 85.7% of SCIWORET cases is notably higher than the 65%-70% reported in previous studies [5,6]. This high prevalence emphasizes the critical role of pre-existing stenosis in SCIWORET pathogenesis, as the narrowed canal creates a vulnerable environment where even minor mechanical stress can trigger SCI [18]. The reduced space available for cord movement in stenotic canals significantly increases susceptibility to neurological damage, particularly in elderly patients, where degenerative changes and vascular compromise may further exacerbate the risk of cord injury under minimal trauma [19,20].

The significant association between cervical OPLL and SCIWORET (OR: 1.83, 95% CI: 1.01-3.29) builds upon previous observations by Chikuda et al. [6], who identified OPLL as a risk factor for acute cervical cord injury. Our results suggest that OPLL contributes to SCIWORET through several interconnected pathophysiological mechanisms [10,21,22]. First, the ossified ligament directly compresses the spinal cord, reducing the effective diameter of the spinal canal. Matsunaga et al. [21] demonstrated that this baseline compression creates a precarious environment where even minor traumatic forces can trigger neurological symptoms. The space available for the spinal cord becomes critically limited, particularly during dynamic movement [23].

Second, OPLL fundamentally alters the mechanical properties of the posterior longitudinal ligament, leading to reduced spinal canal flexibility. This decreased elasticity, as described by Kawaguchi et al. [10], compromises the spine's ability to accommodate mechanical stress during trauma [24]. The normally flexible ligament, which helps absorb and distribute traumatic forces, becomes rigid and potentially transfers these forces directly to the spinal cord. This mechanism is particularly relevant in cases of low-energy trauma, where the altered biomechanics may lead to cord injury despite the absence of obvious fractures.

DISH showed no significant association with SCIWORET in our multivariate analysis (OR: 1.00, 95% CI: 0.59-1.70). While previous studies have focused primarily on cervical DISH, our results align with recent research suggesting that its influence on cervical spine vulnerability may be less direct than previously thought. This could be explained by the biomechanical alterations described by Westerveld et al. [25], including increased spinal column rigidity and modified stress distribution patterns. This discrepancy may be explained by several factors. First, our study specifically examined SCIWORET cases defined by the absence of both bone fractures and DLC injuries on imaging, which differs from the broader definition of cervical SCI used in some previous studies [25,26]. Second, while DISH increases vulnerability to fractures in high-energy trauma due to altered biomechanics and reduced flexibility, it may not significantly influence the specific pathomechanism of SCIWORET, which often occurs in the context of pre-existing canal stenosis.

The complex interplay between DISH, OPLL, and CCS in our multivariate model may also mask individual associations, particularly when these conditions frequently co-exist in elderly patients.

Further studies focusing specifically on the biomechanical effects of DISH in low-energy trauma, and its interaction with other degenerative conditions, such as CCS and OPLL, may help clarify this relationship. Prospective studies with larger sample sizes and detailed biomechanical assessments would be particularly valuable in this regard.

The negative association between high-energy trauma and SCIWORET (OR: 0.72, 95% CI: 0.41-1.29) provides important insights into the pathophysiology of this condition. This finding is consistent with studies by Morishita et al. [18] and Wilson et al. [27], suggesting that SCIWORET may be more common in low-energy trauma situations, particularly in patients with pre-existing cervical pathologies such as CCS and OPLL. The emerging understanding of the relationship between trauma severity and neurological outcomes, in the context of pre-existing cervical pathology, has important implications for clinical practice [18,27].

Our findings regarding the interrelationships between CCS, OPLL, and DISH provide important insights into the pathophysiology of SCIWORET. While these conditions can be clinically overlapping, our multicollinearity analysis indicated that each factor contributes independently to SCIWORET risk. The strong association of CCS with SCIWORET, regardless of OPLL presence, suggests that reduced space available for the spinal cord is a critical pathophysiological mechanism. The analysis of OPLL effects, stratified by DISH status, suggests complex interactions between these ossification disorders that warrant further investigation. Clinically, our findings emphasize the importance of comprehensive radiological assessment in trauma patients to identify these potentially overlapping risk factors for SCIWORET.

Our findings suggest early MRI for trauma patients with neurological symptoms, particularly when CCS or OPLL is suspected. We recommend risk stratification to guide clinical decisions. Emergency departments should implement protocols with lower thresholds for imaging in high-risk patients with CCS and OPLL in order to ensure appropriate specialist consultation, potentially improving early diagnosis and patient outcomes.

This study has several limitations. First, the retrospective design and inclusion of only trauma patients may introduce selection bias. Second, we were unable to assess pre-injury flexibility and balance in these patients - factors that recent research has shown to be potentially important in predicting injury risk. Third, it should be noted that the presence of CCS may represent post-traumatic spinal cord edema rather than a pre-existing anatomical condition, which could potentially confound the interpretation of its role as a risk factor.

## Conclusions

This study provides novel insights into the factors associated with SCIWORET in patients. Our findings suggest that CCS is the strongest associated factor of SCIWORET, regardless of the presence or absence of OPLL. The reduced space available for the spinal cord in patients with CCS may increase vulnerability to neurological injury, leading to an elevated risk of SCIWORET, particularly in low-energy trauma situations. These results emphasize the importance of comprehensive spinal evaluation in trauma patients, especially those presenting with neurological deficits following minor trauma. Future prospective studies are needed to further elucidate the complex relationships between these factors and to develop targeted prevention and management strategies for patients at high risk of SCIWORET.
